# Effects on nurses’ quality of working life and on patients’ quality of life of an educational intervention to strengthen humanistic practice among hemodialysis nurses in Switzerland: a protocol for a mixed-methods cluster randomized controlled trial

**DOI:** 10.1186/s12912-018-0320-0

**Published:** 2018-11-21

**Authors:** Philippe Delmas, Louise O’Reilly, Chantal Cara, Sylvain Brousseau, Jean Weidmann, Delphine Roulet-Schwab, Isabelle Ledoux, Jérôme Pasquier, Matteo Antonini, Tanja Bellier-Teichmann

**Affiliations:** 1 La Source, School of Nursing, University of Applied Sciences of Western Switzerland, Lausanne, Switzerland; 2 Université de Sherbrooke, Sherbrooke, Canada; 3 Université de Montréal, Montreal, Canada; 4 Université du Québec en Outaouais, Saint-Jérôme, Canada; 5 School of Management and Engineering Vaud, Yverdon-les-Bains, Switzerland; 6 Institute of Social and Preventive Medicine, Lausanne University Hospital (CHUV), Lausanne, Switzerland

**Keywords:** Educational intervention, Humanistic nursing practice, Watson’s theory of human caring, Mixed method design, Quality of working life of haemodialysis nurses, Quality of life of haemodialysis patients, Team cohesion

## Abstract

**Background:**

Humanistic nursing practice constitutes the cornerstone of the nursing profession. However, according to some authors, such practice tends to fade over time in favour of non-humanistic behaviours. To contrast this tendency, an educational intervention (EI) based on Watson’s Theory of Human Caring was developed and tested in two pilot studies involving, respectively, rehabilitation nurses in Quebec (Canada) and haemodialysis (HD) nurses in Switzerland. In light of the positive results obtained in these, another study is being undertaken to examine more in depth the EI’s effects on both HD nurses and patients in French Switzerland. The EI is expected to have positive effects on quality of nurse-patient relationship (NPR), team cohesion, nurse quality of working life (QoWL), and patient quality of life (QoL).

**Methods/design:**

The study described in this protocol will use a mixed-method cluster randomised controlled trial design. For the quantitative component, nurse and patient data will be collected through questionnaires. The accessible population of 135 nurses and 430 patients will be clustered into 10 HD units. These units will be randomised into an experimental group (EG) and a waiting-list control group (WLCG). Measurements will be taken at baseline (pre-intervention) and repeatedly over time (post-intervention): immediately at EI completion and six and 12 months thereafter. For the qualitative portion of the study, 18 semi-structured interviews will be conducted with EG nurses picked at random two months after EI completion to explore perceived changes in nurse humanistic practice. Qualitative data will be analysed through the relational caring inquiry method, a phenomenological approach. Descriptive and inferential statistics will be computed from the quantitative data.

**Discussion:**

The study described in this protocol will determine if and how the proposed EI promotes humanistic nursing practice and how this practice affects quality of NPR, nurse QoWL, and patient QoL. Moreover, it will lay the groundwork for offering the EI to nurses in other healthcare sectors.

**Trial registration:**

This clinical study was registered with ClinicalTrials.gov [NCT03283891, 14/09/2017].

## Background

Humanistic nursing practice, including caring, has been a matter of interest to nursing researchers for several decades now. Their studies of the subject have tended to underscore what distinguishes nursing from the other health professions. In this regard, Watson [1] stated that the Theory of Human Caring could serve to guide clinical nursing practice by enabling it to transcend the physical dimensions of the care recipient in order to grasp the whole of the care situation as experienced by the person. More specifically, Watson [1] defines nursing care as helping people give meaning to their existence, suffering, and disharmony by means of a caring relationship. The relationship that nurses cultivate with patients and their families corresponds to a human process that Watson [1] calls the “transpersonal caring relationship”. This is a genuine relationship that requires nurses to base their practice on a system of humanistic-altruistic values that allows patients and their families to grow in an environment conducive to the development of potential [1]. Caring rests on humanistic values that influence attitudes, which in turn guide behaviours. This results in a more humanistic professional practice [1]. According to Cara [2], the attitudes required for nurses to engage in such practice can be developed and strengthened. These include authentic presence, compassion, active listening, understanding, support, reciprocity, and collaboration.

Various authors have documented the therapeutic effects of the caring relationship both on nurses [3], in terms of improved sense of self-esteem, well-being, and personal achievement, and higher levels of work satisfaction [4, 5], and on patients, in terms of improved autonomy, independence, hope [6], and quality of life (QoL) [7], and greater satisfaction with nursing care received [8]. Empirical studies have also shown that the caring relationship contributes to develop a sense of security among patients [5] and to lower hospital readmission rates, in particular among patients with heart failure [9].

Other studies [10–16] have raised awareness of the presence of dehumanising nursing care practices in clinical settings, the effects of which can be devastating for nurses and patients alike. According to the results of a literary meta-analysis by Swanson [14], patients confronted with nurse uncaring attitudes and behaviours feel humiliated, frightened, powerless and vulnerable, and this can contribute to lengthen physical healing times. Moreover, uncaring practices [14] can, for the nurses themselves, lead to burnout and depression and generate the impression of working like an automaton. Though the majority of these studies have been carried out in the United States, similar results have been reported recently in European countries and Canada. In a phenomenological study involving 23 rehabilitation inpatients in Quebec aimed at exploring the meaning and contribution of “being with” a nurse, O’Reilly et al. [17] highlighted various dehumanising elements in nursing practice, including lack of listening, understanding and the objectification of patients. In France, a vast patient-initiated survey on the consequences of kidney disease was undertaken in 2012–2013 within the framework of the “États généraux du rein” [convention of kidney health stakeholders; http://www.renaloo.com/images/stories/EGR/rapport%20final.pdf], in which some 7000 patients undergoing haemodialysis (HD) completed a questionnaire. The results obtained indicated that uppermost among proposed areas of improvement was the quality of the nurse-patient relationship (NPR). The patients underscored that attending staff should demonstrate empathy and a willingness to listen to them and should take the time to explore their needs, which was not always the case.

Against this background and with a view to reviving nursing humanistic practices in care settings, an educational intervention (EI) was developed in Quebec [18, 19] on the basis of Watson’s Theory of Human Caring [20, 21]. The intervention was implemented and evaluated in two pilot studies, first, qualitatively in a population of rehabilitation nurses in Quebec [19] and, then, with mixed methods in a population of HD nurses in French Switzerland [22]. The choice of these two populations rested on the fact that nurse presence and quality of nurse-patient interactions play a key role in patient QoL [23, 24]. Both studies [19, 22] showed that the EI had a high acceptability and feasibility level. Furthermore, a secondary qualitative analysis of the two pilot studies [25] showed that results converged in large part regarding the intervention’s feasibility, acceptability and benefits for nursing practice. This convergence justifies the implementation now of a large-scale experimental study [26, 27] to test the effects of the EI on various outcome variables, including quality of working life (QoWL) of nurses, QoL of patients in their care, and quality of NPR.

### Theoretical framework

In order to enhance the validity of the research through the judicious choice of a theoretical framework [28] to orient the selection of the variables to explore, we propose grounding the study described in this protocol in Duffy and Hoskins’ Quality-Caring Model [9], a middle-range theory that serves to understand the NPR as theorised on the basis of Watson’s 10 carative factors (CF) [20, 21] and to interpret the emergence of positive outcomes for patients, families, health professionals and, more broadly, the healthcare system [29]. According to this model, the NPR is therapeutic for patients by helping raise their QoL and their satisfaction with care. Thus, quality of NPR is a major factor in patient health and an indicator of care quality and patient safety [9]. According to Donabedian’s quality paradigm [30], Duffy and Hoskins’ model [9, 29] can be broken down into three parts: structure, process and outcome. **Structure** includes all factors related to nurses, patients and the healthcare system upstream from care delivery. Where nurses are concerned, these encompass sociodemographic characteristics (e.g., age, years of experience, educational level, shift) and workload-related variables (e.g., patient-nurse ratio, overtime hours worked, team cohesion). For the purpose of our study, we will measure nurse-perceived quality of NPR and QoWL prior to the intervention. As for the patients, we will document sociodemographic characteristics, severity of illness, and comorbidity. We will also measure patient-perceived quality of NPR and QoL. These variables are compatible with the elements that compose Duffy and Hoskins’ model [9, 29]. The measures taken prior to the intervention (pre-test) will provide baseline values. **Process** [9, 29] refers to nurse clinical practice, especially in connection with the caring relationship that nurses cultivate with care recipients. The caring relationship is at the heart of the process and reflects nurse commitment to caring for patients. It is in this light that the relevance of delivering an EI based on the concepts of Watson’s theory [20, 31] becomes clear, given that the intervention is intended to mobilise and consolidate nurse abilities to dispense care according to the caring approach. This is all the more important in HD units where nursing practice revolves around tech-heavy tasks [24]. **Outcome**, the last component of the theory, comprises two levels: intermediate and terminal. For Duffy and Hoskins [9, 29], **intermediate outcomes** correspond to changes in attitudes/behaviours among carers, patients or an organisation, which lead to changes in the orientation of the clinical follow-up of patients. In our study, the following intermediate outcomes will be examined: 1) nurse-perceived changes in caring attitudes/behaviours and patient-perceived changes in nurse caring attitudes/behaviours post-intervention; 2) nurse-perceived changes in team cohesion post-intervention; and 3) quality of NPR. To this end, nurses and patients will complete measures of quality of NPR post-intervention. Moreover, phenomenological qualitative interviews will be conducted with nurses to obtain additional information on their perception of change in quality of NPR post-intervention. Finally, **terminal outcomes** are tied to the major concepts that can affect the future of both HD patients and nurses. In our study, patient QoL and nurse QoWL will be the primary outcomes of the study. In sum, the Quality-Caring Model [9, 29] will provide a solid theoretical structure for examining the effects of the intervention on both the nurses and the patients participating in the research.

### Aim of research and objectives

The aim of the study described in this protocol is to evaluate the effects on quality of NPR, team cohesion, nurse QoWL, and patient QoL of an EI intended to reinforce the humanistic practices of nurses in French-Swiss HD units.

#### Primary objectives

1) Describe levels of the variables of interest among nurses (quality of NPR, team cohesion, QoWL) and patients (quality of NPR, QoL) pre-intervention (T0); and 2) examine the effects of the intervention on the variables of interest among participating nurses and patients immediately post-intervention (T1) and twice more over time (T3 and T4).

#### Secondary objectives

1) Explore and describe nurse-perceived changes in caring attitudes/behaviours post-intervention (T2); and 2) examine the mediating effects of team cohesion on quality of NPR and nurse QoWL.

#### Proposed hypotheses

Based on Duffy and Hoskins’ framework [9, 29] and in accordance with the main objectives of this research, we will make the following statistical hypotheses: 1) post-intervention, frequency of nurse caring attitudes/behaviours will be higher in the experimental group (EG) vs a waiting-list control group (WLCG); 2) post-intervention, team cohesion level will be higher in the EG vs the WLCG; 3) post-intervention, level of nurse QoWL will be higher in the EG vs the WLCG; 4) post-intervention, patient-perceived frequency of nurse caring attitudes/behaviours will be higher in the EG vs the WLCG; and 5) post-intervention, level of patient QoL will be higher in the EG vs the WLCG.

## Method

### Study design

Given the research questions and the intervention’s implementation in a natural setting, we will conduct a mixed-method cluster randomised controlled trial (RCT) [32] with a WLCG. This design allows collecting both quantitative and qualitative data [33]. However, given the aim and context of the study described in this protocol, the quantitative approach will dominate and the qualitative phenomenological approach will be enacted two months post-intervention (T2) to explore process and context factors encountered during the trial. We wish to obtain nurses’ perceptions regarding potential changes after the EI and of potential benefits. The reasons for going with this particular design are multiple. First, the choice of a cluster RCT over a classic RCT is called for given the high risk of contamination between control group and EG if the two are present in the same establishment [34]. Second, mixed-method data collection is recommended particularly when research questions have multiple facets and one method alone cannot serve to explore them all [33], which is the case with our study. Moreover, according to Hesse-Biber [32], incorporating qualitative data collection in a quantitative experimental research design can enhance the findings’ credibility. The choice of a WLCG, for its part, rests on three key factors. First, a higher inclusion rate can be reached if all HD units receive the intervention, whether immediately or one year later. Second, it allows diminishing bias related to prior acquaintance with patients. Third, from an ethical point of view, it seems appropriate for all of the participating nurses to receive the intervention, as it could be beneficial both to their QoWL and to the QoL of patients in their care.

Because of the nature of the EI, nurses cannot be blinded. Researchers are not blinded as well. Nevertheless, a separed group of researcher who are not involved in the EI will conduct data entry and analysis.

### Participants

The studied population is twofold: nurses working in HD units and HD patients in direct contact with these nurses. The nurse population will consist of all nurses working in the HD departments of ten hospitals in the French-Swiss cantons included in the study described in this protocol. This corresponds to an accessible population of 135 nurses. The inclusion criteria will be the same as in the Quebec pilot study [6]: 1) at least 6 months’ work experience in the department; and 2) consent to take part in the study. In addition, nurses who intend to leave the HD unit within three months will be excluded. The patient population will consist of the patients at the HD units participating in the study. This corresponds to an accessible population of 430 patients. Their inclusion criteria will be the following: 1) at least 18 years of age and under active treatment for at least six months; 2) fluency in French; and 3) capacity to provide informed consent. Their exclusion criteria will be the following: 1) diagnosed with dementia; and 2) new to the HD unit. In this regard, six months will be the minimum required period of exposure to the team. As care teams are generally well acquainted with the patients in their care, they will be asked to help with recruitment in order to avoid soliciting patients unable to provide consent and to ensure inclusion criteria are met. Exclusion criteria will be reduced to a minimum to avoid patients feeling snubbed by the care team. Based on past experience, the average participation rate among HD patients is approximately 50% of those who meet the inclusion criteria [25, 35].

### Sampling procedure

For the quantitative component of the study described in this protocol, a cluster will be composed of the nurses and patients of a HD department. The HD units of every hospital in French Switzerland will be contacted to take part in the study, for a total of 13 units. To date, 10 units have already expressed their desire to take part in the study: Centre Hospitalier Universitaire Vaudois (Lausanne), Ensemble Hospitalier de la Côte (Morge), Établissements Hospitaliers du Nord Vaudois (Yverdon), Hôpital du Valais (three independent sites in Sion, Sierre, and Martigny), Hôpital du Jura (Delémont and Porrentruy), Hôpital Intercantonal de la Broye (Payerne), Hôpital Riviera-Chablais (Monthey), Hôpitaux Universitaires de Genève (Geneva).

Once the HD units and all the nurses and patients that compose them are enrolled, they will be randomised by computer to the EG or the WLCG. Among the 1024 (=2^10^) possible allocations to EG and WLCG, we will limit our choices to those where both the EG and the WLCG contain at least 50 nurses. Under this constraint, there are still 410 possible allocations and the number of units per group ranges from 2 to 8. The R software (version 3.3.0) [36] will be used to choose an allocation at random from among these 410 possibilities. All units will have the same probability of being included in the EG.

For the qualitative component, a purposive sample will be used. This sampling method allows selecting persons that have lived the experience under study (in our case, participation in the EI) and that agree to share it. It is best with this method to recruit a wide variety of participants in order to collect as diverse a set of viewpoints as possible on the phenomenon under study. Participants for this component will be selected from among the EG nurses. Moreover, in order to take account of staff heterogeneity across EG units, the number of participants invited to be interviewed will be weighted based on unit size. It is difficult with qualitative approaches to determine beforehand the number of participants required. Benner [37] recommends 5 to 25 but specifies that it is necessary to keep interviewing until the discourse becomes redundant, that is, until adding participants to the sample and more data to those already collected sheds no more light on the phenomenon under study [38]. Based on the experience of researchers in the field, it is estimated that a sample of 18 participants will be required.

### Intervention description and delivery

The EI was originally developed with rehabilitation nurses working with spinal cord injury patients by Quebec researchers [19, 35] guided by Watson’s Theory of Human Caring [20, 21, 31]. The researchers, experts in humanist philosophy, focused on the choice and definition of the theoretical concepts to be taught and on the creation of learning activities [19] (see Table 1) deemed indispensable for optimal concept appropriation and application within the clinical care context of spinal cord injury rehabilitation (Quebec) and, later, haemodialysis (Switzerland). In Switzerland, the EI will be delivered by the two principal researchers of the study described in this protocol. Each has substantial clinical experience with persons living with a chronic condition and has previously taught Watson’s Theory of Human Caring [20, 21]. The EI will be delivered to all the nurses participating in the study but at different times depending on group assignment (see Table 2). Intervention delivery schedules will be specific to each centre based on availability within work schedules. In order to facilitate learning and interactions during sessions, groups will comprise 3 to 8 people. Each group will complete all four sessions, each lasting 3.5 h. There will be a minimum interval of one week between sessions to afford participants the necessary time to reflect on the content, to appropriate it and to apply it in their clinical practice. The intervention will be implemented in the training rooms of the establishments partnering in the research project.

**Table 1:** Processes and content of educational intervention

Session and topic	Objectives	Learning activities
Session #1: Acquisition of core concepts of caring practice	- Caring values, attitudes and behaviours - Describe core concepts of Watson’s Theory of Human Caring ▪ Phenomenal field ▪ Transpersonal caring relationship ▪ Caring occasion and caring moment ▪ Moral ideal - Develop concept of “phenomenal field” further - Recognise concepts taught in caring reflective clinical vignette #1 - Gain awareness of relevance of humanistic approach to caring for HD patients - Present a schematic representation of theoretical concepts	- Print documents and PowerPoint presentation - Print article on theory [38] - Focusing exercise - Theory course - Practical listening exercise - Narrative #1: Roger on concept of phenomenal field - Plenary discussion - Schematisation of theoretical concepts [57] - Reflective assignments (1) Read caring reflective clinical vignette #1: The caring moment: a moment of well-being for both patient and nurse (2) Answer reflective questions
Session #2: Acquisition of coreconcepts of caring practice	- Describe concept of Watson’s 10 carative factors and give clinical example for each - Integrate concept of 10 carative factors further - Recognise concepts taught in caring reflective clinical vignette #2 - Present a schematic representation of theoretical concepts	- Print documents and PowerPoint presentation - Review of session #1 (content and clinical vignette) and questions - Focusing exercise - Narrative #2: Roger on changing phenomenal field and carative factors - Theory course - Exercise on identifying nurse and patient resources [58, 59] - Group discussion - Schematisation of theoretical concepts [57] - Reflective assignments (1) Read caring reflective clinical vignette #2: Nursing interventions guided by carative factors to comfort Simone (2) Answer reflective questions (3) Propose resource exercise to a HD patient and do a summary
Session #3: Appropriation of concept of hope Incorporation of all concepts taught	- Describe different attributes of concept of hope - Gain awareness of what hope can contribute to HD patient QoL - Recognise different humanistic interventions that influence hope in caring reflective clinical vignette #3 - Discuss search for meaning in HD patients - Present schematic representation of theoretical concepts	- Print documents and PowerPoint presentation - Review of session #2 (content, clinical vignette and exercise to identify resources with a patient) and questions - Focusing exercise - Narrative #3: Roger on changing phenomenal field and nursing interventions to strengthen hope - Theory Course - Group discussion - Schematisation of theoretical concepts [57]. - Reflective assignments (1) Read: Caring reflective clinical vignette #3: Inspiring hope like a breath of life (2) Answer reflective questions
Session #4: Enactment of different caring attitudes and behaviours in an intermediary simulation exercise with professional actor	- Mobilise concepts of Watson’s Theory of Human Caring in a safe environment where mistakes are permitted (simulations) - Foster connections between knowledge acquired and HD clinical practice	- Print documents - Review of session #3 (content and clinical vignette) and questions - Two simulation exercises comprising: *briefing:* reading of first clinical situation and choice of nurse for simulated practice; *simulated practice:* simulation with nurse and actor according to predetermined scenario for actor; *debriefing:* sharing of experiences (nurse, actor and group); remobilisation, deeper understanding and further development of theoretical concepts

**Table 2:** Data collection over time for nurses and patients

		Enrolment	Allocation	Post-allocation				Close-out	Post-trial
TIME POINT		t-_1_	t_0_	Intervention	t_1_ immediately post-intervention	t_2_ 2 months post-intervention	t_3_ 6 months post-intervention	t_4_ 12 months post-intervention	Intervention
ENROLMENT	Project presentation to nursing care directors at different sites	x							
	HD units recruited	x							
	Informed consent obtained for HD nurses and patients		x						
	Cluster randomisation		x						
INTERVENTION	Experimental group (EG)			➨					
	Waiting-list control group (WLCG)								➨
ASSESSMENT									
HD nurses (EG and WLCG)	QUANTITATIVE DATA								
	Sociodemographic questionnaire		x						
	CNPI-70/EIIP-70		x		x		x	x	
	GEQ		x		x		x	x	
	QoWL		x		x		x	x	
	Team cohesion		x		x		x	x	
HD nurses (EG)	QUALITATIVE DATA								
	Qualitative interviews					x			
HD patients (EG and WLCG)	Sociodemographic and medical questionnaire		x						
	CNPI-70/EIIP-70		x		x		x	x	
	WHOQOL-BREF		x		x		x	x	

### Quantitative data collection

Various instruments will be used to collect data from participating HD nurses and patients.

### HD nurses

#### Sociodemographic data

At pre-test (T0), personal data (sex, age) and work-related information (employment status, years of experience as HD nurse, number of patients attended to per work shift) will be collected.

The nurse-patient relationship (NPR) will be measured with the French-language version (EIIP-70) [39] of the Caring Nurse-Patient Interaction Scale (CNPI-70). This tool comprises 70 items covering 10 dimensions that capture each of Watson’s 10 CF. It allows nurses to self-rate their frequency of caring attitudes/behaviours. The dimensions covered by the instrument are the following [39]: humanism (6 items), hope (7 items), sensitivity (6 items), relationship (7 items), emotions (6 items), problem solving (6 items), teaching (11 items), assistance (10 items), and existential factors (6 items). Respondents must choose from five responses ranging from 1 (almost never) to 5 (almost always). This tool was validated on 377 nursing students and demonstrated good psychometric properties (range of Cronbach’s alphas for the 10 sub-dimensions of 0.73 to 0.91) [39]. Moreover, the two pilot studies showed respondents had no difficulty understanding the questionnaire and completing all the questions.

Team cohesion will be measured with the Group Environment Questionnaire (GEQ) [40] adapted for work teams [41]. A French-language version of the questionnaire was created following the back-translation procedure [41]. Like the original, the French-language version comprises 18 items and encompasses the four dimensions of team cohesion [42], namely, respondent social-related attraction to group (five items, one of which is reverse scored), respondent task-related attraction to group (four items, two of which are reverse scored), social-related group integration as perceived by respondent (four items, two of which are reverse scored), and task-related group integration as perceived by respondent (five items, one of which is reverse scored). The response scale ranges from 1 (strongly disagree) to 5 (strongly agree). The questionnaire was tested on a sample of nurses from another healthcare institution. No major comprehension difficulties emerged. Cronbach’s alphas for the different dimensions ran from 0.71 to 0.81 [41].

Quality of working life (QoWL) will be measured with a scale developed by Elizur and Shye [43], translated into French and validated by Delmas, Escobar, and Duquette [44]. The scale is composed of 16 items exploring four dimensions of QoWL [43]: psychological (items 1–4), physical (items 5–8), social (items 9–12), and cultural (items 13–16). Respondents must choose from six answers on a Likert scale ranging from “a very large part” to “very little”. The scale’s empirical structure was tested on a population of 540 Hungarian industrial workers [43]. The internal consistency of the overall scale was estimated at 0.90 (Cronbach’s alpha). Also, Delmas, Escobar, and Duquette [44] obtained Cronbach’s alphas of 0.87 to 0.89 for its various dimensions with a population of French nurses.

### HD patients

The patients will complete a sociodemographic and medical questionnaire. It will be based on elements selected by Boini et al. [45] for the purposes of a longitudinal study of the QoL of 1000 French chronic kidney disease patients. It will comprise several sections covering sociodemographic characteristics (age, sex, marital status, number of children, income), clinical status, and bio-clinical data (weight, size, number of drugs taken daily, alcohol and tobacco use, albuminemia level, haemoglobin level), comorbidity (cardiovascular disease, diabetes, hypertension), and disabilities (amputation of lower limbs, para/hemiplegia, blindness). This questionnaire presented no particular problems when previously tested on Swiss HD patients [6, 46].

The nurse-patient relationship (NPR) will be explored with the French-language version (EIIP-70) [3] of the CNPI-70 (for a description, see *Nurse* section above). The items were adapted to the patient’s viewpoint merely by changing the person addressed. The scale presented perfectly satisfactory psychometric properties (Cronbach’s alphas of 0.75 to 0.85) when validated on a French population of patients living with HIV [47]. The scale allows HD patients to assess nurse caring attitudes and behaviours.

Quality of life (QoL) of HD patients will be explored with the WHOQOL-BREF. This scale is a generic instrument that serves to explore perceived QoL [48] and impact of health problems on QoL [49]. It comprises 26 items exploring four dimensions: physical health (7 items), psychological health (6 items), social relationships (3 items), and environment (8 items). It also contains two items measuring overall QoL, which is an added advantage of this instrument. Respondents must choose from five responses on a Likert scale of 0 (not at all) to 5 (completely). The WHOQOL-BREF yields an overall “QoL profile” score ranging from 0 to 100, as well as scores for each dimension. A high score indicates a high perceived QoL [48, 49]. The English-language version of the instrument has shown good content and discriminant validity, as well as excellent internal consistency [48]. The French-language version translated and validated by Leplège et al. [50] has shown perfectly satisfactory psychometric properties (Cronbach’s alphas of 0.82, 0.81, 0.68, and 0.80, respectively, for the above dimensions) and good acceptability by the population, with a non-response rate below 5%. When the questionnaire was administered in the pilot study [35] conducted in Switzerland, HD patients had no difficulty completing the questionnaire.

### Qualitative data collection

Qualitative data will be collected through a phenomenological method, the Relational Caring Inquiry, developed by Cara [51, 52] on the grounds of Watson’s work and humanist philosophy. The method entails seven inter-related dynamic phases: 1) acknowledging researcher’s worldview; 2) seeking participants; 3) being present for participants’ stories; 4) discovering essence of participants’ stories; 5) reciprocating participants’ stories; 6) relational caring process; and 7) elucidating essence of phenomenon [53]. An interview guide has been developed by the investigators to enhance the rigour of qualitative data collection. The aim of the phenomenological qualitative approach used is to gain a deeper understanding and description of nurse perceptions regarding: 1) post-intervention practice transformation; 2) post-intervention teamwork cohesion transformation; and 3) intervention benefits for themselves and HD patients. Data will be collected by way of semi-structured interviews recorded at the EG nurses’ workplace, in a place providing privacy, where a relationship of mutual trust can be cultivated and confidentiality ensured. Finally, to ensure the rigour of the qualitative data collection process, the investigators will keep a logbook for tracking purposes, in which they will jot down methodological notes (e.g., events in development/evolution of method, changes), theoretical notes (e.g., interpretations, deductions, hypotheses, theoretical knowledge), and personal notes (e.g., feelings, emotions, impressions).

### Data analysis

For the quantitative component of the study described in this protocol, first, we will check the data for inclusion conditions and outliers. Outlying data will be verified individually. Second, associations between factors and outcomes will be described using frequencies, means and standard deviations or medians and interquartile ranges. In addition, these associations will be evaluated using chi-square tests, Fisher tests or analyses of variance. Third, linear regression models adjusted for significant sociodemographic and health variables will be used to examine the intervention’s effect on the different variables selected for the study, keeping in mind that nurse QoWL remains the key outcome variable. Moreover, we will consider a random intercept to take HD unit variance into account. Fourth, we will test whether team cohesion acts as a mediator in the relationship between NPR (independent variable) and nurse QoWL (outcome variable). For this purpose, we will run three regressions: 1) regression of mediator on independent variable (EIIP-70); 2) regression of outcome variable (QoWL) on independent variable; and 3) regression of outcome variable on both independent variable and mediator. Fifth, an intention-to-treat analysis will be conducted of the data on all participants, whether they complete the intervention or not. This will diminish bias related to protocol deviations or withdrawals and will allow estimating the intervention’s effects under conditions that are close to real life. Sixth, based on sample size and sample design (10 HD units of different sizes for a total of 135 nurses) and considering an intra-class correlation coefficient of 0.03 for the HD units, we will be able to detect an effect size of 0.6, with statistical power of 0.8 and significance level at 0.05 for all analyses. The expected effect size has been fixed by the investigators and corresponds to the minimal clinically significant effect. The intra-class correlation coefficient selected is in line with Giraudeau’s recommendations [52] of 0.001 to 0.05 for a cluster RCT. The data will be analysed using the R software [54].

The qualitative data will be organised using NVivo software (version 11). All of the interviews will be transcribed verbatim. Following the RCI phenomenological method [51, 52], transcripts will be analysed to identify units of meaning (first-level reflection), that is, ideas specifically regarding the phenomenon under study. Next, the units will be grouped into sub-themes (second-level reflection), which correspond to descriptive elements that reflect as best possible the participants’ transcripts. Then, the sub-themes will be organised into themes (third-level reflection) based on a convergence of ideas [52]. Thereafter, an in-depth analysis of these themes, as well as use of free imaginary variation, will allow us to identify the essential constructs of the phenomenon, that is, the eidos-themes (fourth-level reflection) [52]. Finally, these eidos-themes will allow the universal essence of the phenomenon under study to emerge (fifth-level reflection) [51, 52]. The scientific validity standards that apply to qualitative research, namely, authenticity (confirmability), credibility, criticality (dependability), integrity and transferability (fittingness) [55], will be a constant consideration for the researchers.

Convergence between the quantitative and qualitative results will then be analysed by comparing and discussing the two using a side-by-side approach [33, 56]. The aim here will be to determine the extent to which the results derived from the two types of data converge.

### Ethical considerations

Special attention will be focused on three ethical elements: informed consent (of nurses and patients), confidentiality, and support. Regarding informed consent, the study participation form will provide information on the purpose of the study described in this protocol, the measures to be used to ensure anonymity, and freedom to withdraw from the study at any time without consequence for their professional career in the case of the nurses and for treatment and care received in the case of the patients. Consent forms completed by respondents will be kept in a suitable place for as long as required by the Swiss Ethics Committees on Research Involving Humans. Regarding confidentiality, the names of the respondents will not appear on any form or interview transcript. A code will be assigned to each form and a letter to each establishment. For the purposes of longitudinal follow-up, a password-protected file will be created containing the information necessary to administer questionnaires post-intervention. This file will be destroyed upon completion of the repeated measurement at twelve post-intervention. Regarding support, respondents will be able to reach a contact person throughout the study if they have any questions. They will also be able to access all the study results and/or a summary of the highlights by email, telephone, conventional post or the Moodle interface. The principal investigator will be responsible for drafting the final research report. Researchers have already obtained the required ethics certificate from the Human Research Ethics Review Board of the Canton of Vaud (Switzerland).

### Preliminary results

The inclusion of participants was initiated in September 2017. Currently, we have completed the first wave of data collection, including 140 patients and 100 nurses. The EI has been deployed to all the nurses included in the EG.

## Discussion

Though Watson’s Theory of Human Caring has often been described as a vital guide to humanistic nursing practices, to date, very few studies in the field of nursing have sought to test this disciplinary theory experimentally. Such research is essential as the results obtained would serve to consolidate the theory’s construction and to guide us in improving the EI. By examining the EI’s beneficial effects on both nurses and the HD patients in their care, the study described in this protocol will allow us to gain a better understanding of the EI’s direct effects on nurses’ clinical practice transformation and its indirect effects on HD patients’ QoL over the long term. The results obtained will allow us to bolster the relevance of humanistic practice as a means of ensuring quality of care and patient safety. Continuing professional development activities need to be offered to nurses to this end. The study described in this protocol will help us determine whether the EI in question is effective in this regard.

### Limitations

We are opting for a cluster RCT for fear of contamination between the EG and the WLCG should the two be present in the same unit. As with a conventional RCT, numerous potential factors could compromise the validity of the study described in this protocol and thus bias the results. Uppermost among these are the high risk of participant dropout, among both nurses and patients, owing to the study’s longitudinal design. In order to limit this potential bias, an information letter will be created to keep participating nurses abreast of how the research project is progressing. Moreover, regular contacts between the research team and the head nurses of the different units included in the study will be planned over the entire course of the project. Where the HD patients are concerned, each data collector will take the time to read the questionnaire comprising the different instruments of measure to each patient who manifests the desire to take part in the study. Such a strategy proved effective in the past when used by the principal investigator to collect data from this population [35]. In addition, the research team could have some difficulty implementing the EI if participation translated into a heavier workload for participating nurses. In order to limit this potential bias, all the partnering care institutions will consider the time spent by the nurses engaging in the EI as work time. Also, the institutions will consider the EI as top-priority training for HD units.

## Abbreviations

CF: Carative Factors
CNPI-70: Caring Nurse-Patient Interaction Scale
EG: Experimental Group
EI: Educational Intervention
EIIP-70: French-language version of the Caring Nurse-Patient Interaction Scale
GEQ: Group Environment Questionnaire
HD: Haemodialysis
NPR: Nurse-Patient Relationship
QoL: Quality of Life
QoWL: Quality of Working Life
RCI: Relational Caring Inquiry
RCT: Randomised Controlled Trial
WHOQOL-BREF: World Health Organization Quality of Life scale (short version)
WLCG: Waiting-List Control Group
